# Antifouling and Mechanical Properties of Photografted Zwitterionic Hydrogel Thin-Film Coatings Depend on the Cross-Link Density

**DOI:** 10.1021/acsbiomaterials.1c00852

**Published:** 2021-08-04

**Authors:** Megan J. Jensen, Adreann Peel, Ryan Horne, Jamison Chamberlain, Linjing Xu, Marlan R. Hansen, C. Allan Guymon

**Affiliations:** † Department of Otolaryngology—Head & Neck Surgery, University of Iowa, Iowa City, Iowa 52242, United States; ‡ Department of Chemical and Biochemical Engineering, University of Iowa, Iowa City, Iowa 52242, United States; § Department of Neurosurgery, University of Iowa, Iowa City, Iowa 52242, United States; ∥ Department of Molecular Physiology and Biophysics, University of Iowa, Iowa City, Iowa 52242, United States

**Keywords:** zwitterionic polymer, foreign body response, biofouling, biomaterial coatings, photografting, polymer thin film

## Abstract

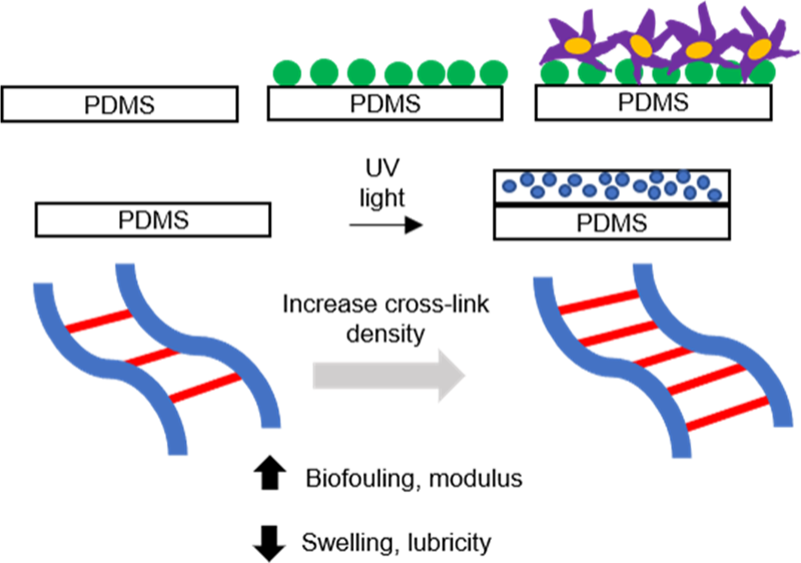

Zwitterionic polymer networks have shown promise in reducing the short- and long-term inflammatory foreign body response to implanted biomaterials by combining the antifouling properties of zwitterionic polymers with the mechanical stability provided by cross-linking. Cross-link density directly modulates mechanical properties (i.e., swelling behavior, resistance to stress and strain, and lubricity) but theoretically could reduce desirable biological properties (i.e., antifouling) of zwitterionic materials. This work examined the effect of varying poly(ethylene glycol) dimethacrylate cross-linker concentration on protein adsorption, cell adhesion, equilibrium swelling, compressive modulus, and lubricity of zwitterionic thin films. Furthermore, this work aimed to determine the appropriate balance among each of these mechanical and biologic properties to produce thin films that are strong, durable, and lubricious, yet also able to resist biofouling. The results demonstrated nearly a 20-fold reduction in fibrinogen adsorption on zwitterionic thin films photografted on polydimethylsiloxane (PDMS) across a wide range of cross-link densities. Interestingly, either at high or low cross-link densities, increased levels of protein adsorption were observed. In addition to fibrinogen, macrophage and fibroblast cell adhesion was reduced significantly on zwitterionic thin films, with a large range of cross-link densities, resulting in low cell counts. The macrophage count was reduced by 30-fold, while the fibroblast count was reduced nearly 10-fold on grafted zwitterionic films relative to uncoated films. Increasing degrees of cell adhesion were noted as the cross-linker concentration exceeded 50%. As expected, increased cross-link density resulted in a reduced swelling but greater compressive modulus. Notably, the coefficient of friction was dramatically reduced for zwitterionic thin films compared to uncoated PDMS across a broad range of cross-link densities, an attractive property for insertional implants. This work identified a broad range of cross-link densities that provide desirable antifouling effects while also maintaining the mechanical functionality of the thin films.

## Introduction

The use of implantable medical devices has dramatically increased over the past several decades. However, the success of these devices is limited by the foreign body response (FBR).^1,2^ Upon implantation of a biomaterial, the FBR begins with the almost immediate adsorption of host blood proteins to the biomaterial surface.^3−7^ This response escalates as inflammatory cells infiltrate the region. Macrophages migrate to the surface of the biomaterial, fuse to form foreign body giant cells, and recruit other inflammatory cells, including fibroblasts, to the site of the implant.^4^ Macrophages and fibroblasts contribute to the formation of the granulation tissue, which ultimately results in the fibrous encapsulation of the implant.^3,4,6^ The FBR and resultant fibrosis can have detrimental consequences, particularly for neural prostheses, including cochlear implants (CIs), which depend on the fine spatial delivery of electrical stimulation to the surrounding nervous tissue.^5,8,9^ The fibrotic capsule increases the electrode impedance for such implants, leading to a significantly decreased performance.

Several strategies have been investigated to modulate the FBR to biomaterials including surface modification, biomimetic materials, and other molecular, pharmacologic, or cell-based strategies.^10^ For example, in the cochlea, delivery of glucocorticoids has been used to reduce inflammation following CI placement.^11−15^ However, this strategy does not provide a sustainable solution for long-term implants with limited drug elution time scales. In other biomaterial applications, hydrophilic and biologically compatible polymers such as poly(ethylene glycol) (PEG) and poly(hydroxyethyl methacrylate) have commonly been used to decrease biofouling.^16^ However, these materials still allow a significant degree of protein adsorption and fibrosis.^17,18^

Surface modification using ultralow fouling zwitterionic polymers, including poly(sulfobetaine methacrylate) and poly(carboxybetaine methacrylate), shows promise in reducing the FBR and fibrosis.^5,9,19−22^ Zwitterionic polymers, which are electrically net-neutral and hydrophilic, recruit a dense hydration layer to their surface, making adsorption of proteins and other molecules energetically unfavorable.^5,17−21,23^ Recently, we have described a photochemical process for simultaneous photopolymerization, photografting, and cross-linking of zwitterionic thin films on relevant implant materials, including polydimethylsiloxane (PDMS).^8,24^ These zwitterionic thin-film coatings demonstrated decreased protein and cellular adsorption, as well as bacterial adhesion.^21,24^

Zwitterionic thin films are often formed with cross-linking molecules that enable the formation of a covalent network that produces mechanically stable and durable zwitterionic polymers.^21^ However, these cross-linkers are typically not zwitterionic. Changing the ratio of cross-linker to zwitterion could therefore significantly alter both the mechanical stability and antifouling properties of the polymer. Ideally, thin-film coatings for biomaterial applications should have a balance of these properties, in which they can resist biofouling while maintaining mechanical stability. Thin films grafted to CIs, for example, have the potential to reduce protein/cell adhesion and subsequent fibrosis. At the same time, these materials should ideally be lubricious to reduce the friction associated with insertion forces, be sufficiently durable to withstand wear and tear of insertion and lifelong use, and reduce any risk of trauma to intracochlear structures through excessive swelling. Each of these properties plays a role in the dynamic function of biomaterial thin films and is likely directly dependent on the cross-link density.

In this work, we aim to create zwitterionic thin films grafted to PDMS that exhibit both robust mechanical (e.g., swelling, stress–strain behavior, and lubricity) and antifouling properties by determining how cross-link density affects these properties. We hypothesize that increasing cross-linking of films will strengthen the films while decreasing swelling, lubricity, and biofouling resistance. This work demonstrates the role of cross-linking in allowing suitable mechanical and biological properties for the development of biomaterial thin-film coatings. An improved understanding of the relationship and balance between these properties is essential for the successful deployment of zwitterionic thin films for biomaterial applications.

## Experimental Section

### Materials

PDMS materials included Sylgard 184 (Dow, Midland, MI) and reinforced medical grade PDMS (0.1 in. thickness, Bentec Medical, Inc., Woodland, CA). Benzophenone, acetone, [2-(methacryloyloxy)ethyl]dimethyl-(3-sulfopropyl)ammonium hydroxide (SBMA), poly(ethylene glycol) methacrylate (PEGMA) with an average molecular weight of 500, paraformaldehyde, collagenase, Dulbecco’s modified Eagle’s medium (DMEM), DMEM/F12, phosphate-buffered saline (PBS), and l-glutamine were obtained from Sigma-Aldrich (St. Louis, MO). 3-{[2-(Methacryloyloxy)ethyl]dimethylammonio}propionate (CBMA) was purchased from TCI Chemicals (Portland, OR). PEG dimethacrylate (PEGDMA) with an average molecular weight of 400 for the PEG block (Polysciences, Warrington, PA) was used as the cross-linking molecule for the zwitterionic thin films. Recombinant antivimentin antibody and anti-F4/80 antibody were obtained from Abcam (Cambridge, MA). DAPI-containing mounting medium, recombinant human granulocyte/macrophage colony-stimulating factor (GM-CSF), fetal bovine serum (FBS), and TrypLE Express were acquired from Gibco (Waltham, MA). 488 goat antirabbit secondary antibody, 546 goat antirat secondary antibody, fluorescently labeled fibrinogen Alexa 546, Irgacure 2959, and glass coverslips were obtained from Thermo Fisher. An Omnicure S1500 lamp (Lumen Dynamics, Mississauga, Canada) was used for photocuring and photografting.

### Methods

#### Production of Zwitterionic Hydrogels on PDMS

Zwitterionic hydrogel coatings were prepared on medical grade PDMS or on Sylgard 184 PDMS substrates. Sylgard 184 PDMS was produced by using 10 parts base to 1 part curing agent on a mass basis, placing under vacuum for 1 h to remove entrapped air, and curing in an oven at 90 °C for 1 h. Samples were then cut to the desired length and width (23 mm × 23 mm).

To prepare for zwitterionic and other polymeric thin-film grafting, PDMS substrates were first soaked in a 50 g/L solution of benzophenone in acetone for 1 h to enable subsequent surface photografting. After removal from the solution, any residual solution was evaporated using a nitrogen gas stream. The functionalized PDMS substrates were then placed under vacuum for 1 h to ensure the complete removal of any remaining acetone. To create grafted hydrogels on PDMS, 20 μL of the monomer solution was pipetted onto the PDMS and dispersed via capillary action across the surface by a 25 mm × 25 mm coverslip. The array was then exposed to 30 mW/cm^2^ of 365 nm UV light measured from a high-pressure mercury bulb. The total monomer concentration was kept at 35 wt % in deionized water with the proportion of PEGDMA to SBMA/CBMA varied from 0 to 100% of the total monomer. Additionally, 0.05 wt % Irgacure 2959 photoinitiator was added. For example, a CBMA monomer solution with 5% cross-linker would have a total mass composition of approximately 0.05 wt % Irgacure 2959, 64.95 wt % distilled water, 1.75 wt % PEGDMA, and 33.25 wt % CBMA.

#### Protein Adhesion Quantification with Variable Cross-Link Densities

To determine the effect of altering cross-link density on protein adhesion, photografted zwitterionic thin films on medical grade PDMS (Bentec Medical, Woodland, CA) were exposed to fluorescently labeled fibrinogen (Thermo Fisher), washed, and then measured for fluorescence. In detail, 30 μL of 1.0 mg/mL fluorescently labeled fibrinogen solution (Alexa Fluor 546) was placed on the films and dispersed by capillary action using a 25 mm × 25 mm coverslip. After 1 h, the films were rinsed three times and then mounted on a 22 mm × 60 mm cover glass for analysis under an epifluorescence microscope (Leica). Nine images per sample were taken and analyzed using ImageJ to measure raw fluorescence. The fluorescence was averaged across samples and normalized to uncoated PDMS.

#### Cell Culture and Density Quantification with Variable Cross-Link Densities

To investigate the impact of altering cross-link density on cell adhesion, macrophages and fibroblasts were cultured on medical grade PDMS coated with zwitterionic thin films with varying cross-linker concentrations, and cell density was measured. Bone marrow-derived macrophages were obtained from 4- to 6-week-old CBA/J mice, as previously described.^25^ The bone marrow progenitor cells were maintained in a macrophage complete medium (DMEM/F12 with 10% FBS, 10 mM l-glutamine, 100 units/mL GM-CSF). Cell suspension (1.5 mL of 1.7 × 10^6^ cells/mL) was seeded onto 7 × 7 mm zwitterionic-coated PDMS substrates in 12-well plates. The cell medium was changed on day 3. The cultures were maintained for 7 days. The cells were then fixed in 4% paraformaldehyde in PBS, followed by cell permeabilization with a blocking buffer (1% BSA, 0.3% Triton-X in PBS). All substrates were then incubated with the anti-F4/80 antibody (Abcam ab6640, 1:200 in blocking buffer), a macrophage marker, for 2 h at 37 °C. After 2 h, the substrates were rinsed twice with PBS and secondary antibody (Alexa 546, Thermo Fisher, 1:400 in blocking buffer) was applied for 1 h at room temperature. Coverslips with DAPI-containing mounting medium were then placed on each substrate for nuclear staining. Cell density was determined using fluorescence microscopy. Ten randomly selected 20× images were obtained per substrate. ImageJ software was used to count cells. The experiment was performed in triplicate.

Fibroblasts from the spiral ligament of the cochlea were dissected from p2-5 CBA/J mice pups.^26^ The spiral ligaments were pooled and digested in equal parts by 0.12% trypsin and 0.2% collagenase for 10 min at 37 °C with intermittent shaking. Trypsin was inactivated with FBS. The cells were rinsed with DMEM, triturated, and seeded onto 35 mm tissue-culture-treated Petri dishes. The fibroblasts were maintained in DMEM 10% FBS and grown until confluent (48–72 h). Once confluent, the fibroblasts were dissociated from the cell culture dish with TrypLE Express for 10 min at 37 °C. After resuspending the cells in DMEM 10% FBS, the cells were plated on zwitterionic-coated substrates in a 12-well plate with the same cross-link densities used above. 1 mL of the cell suspension (2 × 10^4^ cells/mL) was plated onto each substrate. The cultures were maintained for 48 h, then fixed, and immunostained with antivimentin antibody, a fibroblast marker (Abcam ab92547, 1:200), and a secondary antibody (Alexa 488, Thermo Fisher, 1:400). They were then cover-slipped with the DAPI-containing mounting medium (Gibco). Fluorescence microscopy was used to determine cell counts, as mentioned above. The experiment was performed in triplicate.

### Physical Characteristics

Photografted thin films were evaluated by scanning electron microscopy (SEM) and confocal microscopy to ensure consistent surface characteristics, thickness, and quality. Thin-film surfaces were uniform and smooth (see Supporting Information, Figure S1-Sn). Photografted coatings were approximately 40 μm thick when hydrated, as determined by water-tracing confocal microscopy (see Supporting Information, Figure S2-Sn).

To determine the impact of cross-link density on the mechanical properties of the hydrogel, free-standing thin films were examined. Equilibrium swelling, as a function of cross-link density, was measured by creating 1 mm thick zwitterionic polymeric films. After photopolymerization, hydrogels were placed in PBS or FBS solution to more closely represent the physiological conditions and avoid pH changes that can significantly influence the swelling of zwitterionic materials.^24^ The samples were allowed to swell until no further mass increase was observed. The hydrogels were then vacuum-dried for 24 h, and dry mass was recorded. Equilibrium swelling percent was calculated as follows in eq 1
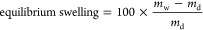
1where *m*_w_ is the mass of the hydrogel sample after swelling to equilibrium and *m*_d_ is the mass after drying.

Compressive modulus was determined using a dynamic mechanical analyzer (DMA Q800, TA Instruments). 1 mm thick hydrogels were prepared and subsequently cut into discs (diameter 1 cm). Prior to testing, samples were swollen to equilibrium in PBS. The hydrogels were tested by the uniform application of compressive stress at a rate of 0.5 N/min to obtain stress and strain curves. The modulus of elasticity was calculated for each hydrogel sample between one and five percent strain using eq 2
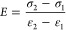
2where *E* is the modulus of elasticity, σ is the stress, and ε is the strain. The subscripts correspond to points at approximately one (subscript 1) and five percent (subscript 2) strain. Each condition was replicated four times, from which the average and standard deviation were calculated.

To measure lubricity, reinforced medical grade 25 mm diameter PDMS discs were prepared using a gasket punch. Discs were coated by photopolymerization and photografting, as described above with CBMA, SBMA, and PEGMA at varying cross-link densities. The coefficient of friction was measured for each sample using a pin-on-disk tribometer (TRB3, Anton Paar). Each sample was mounted in an immersion cup and covered with PBS. The tribometer measured the coefficient of friction using a 6 mm sapphire ball as a probe in the rotational movement mode. The mean coefficient of friction was calculated over 20 laps around each sample. This mean was then normalized to the measured coefficient of friction for uncoated PDMS.

### Statistics

Statistical analysis was performed using GraphPad Prism 8.0. Comparison of protein and cell adhesion on uncoated and SBMA-/CBMA-coated PDMS at varying cross-link densities was performed using one-way ANOVA with post hoc Tukey tests. Mean and standard deviation were calculated to compare material parameters at varying cross-link densities.

## Results and Discussion

### Protein Adsorption

Protein adsorption is the initial step in the FBR with surface protein adsorption modulating the subsequent adhesion of cells to a biomaterial surface.^4^ Thus, the ability of a biomaterial to resist protein adsorption can indicate how well the material will reduce the FBR. Therefore, the adsorption of fibrinogen, a key protein identified in FBR, was examined relative to the cross-link density of grafted zwitterionic thin films. Zwitterionic hydrogels are a class of materials with exceptional resistance to protein adhesion that have outperformed conventional hydrogel materials such as PEG-based polymers.^27,28^ Other work has looked at protein adhesion or accumulation on or within PEG hydrogels as a function of cross-link density.^29,30^ Interestingly, decreasing cross-linking density resulted in an increase in protein accumulation.^30^ As such, it was hypothesized that the cross-linker PEGDMA would increase protein adsorption on a zwitterionic hydrogel in proportion to its percent composition.

**Figure 1:**
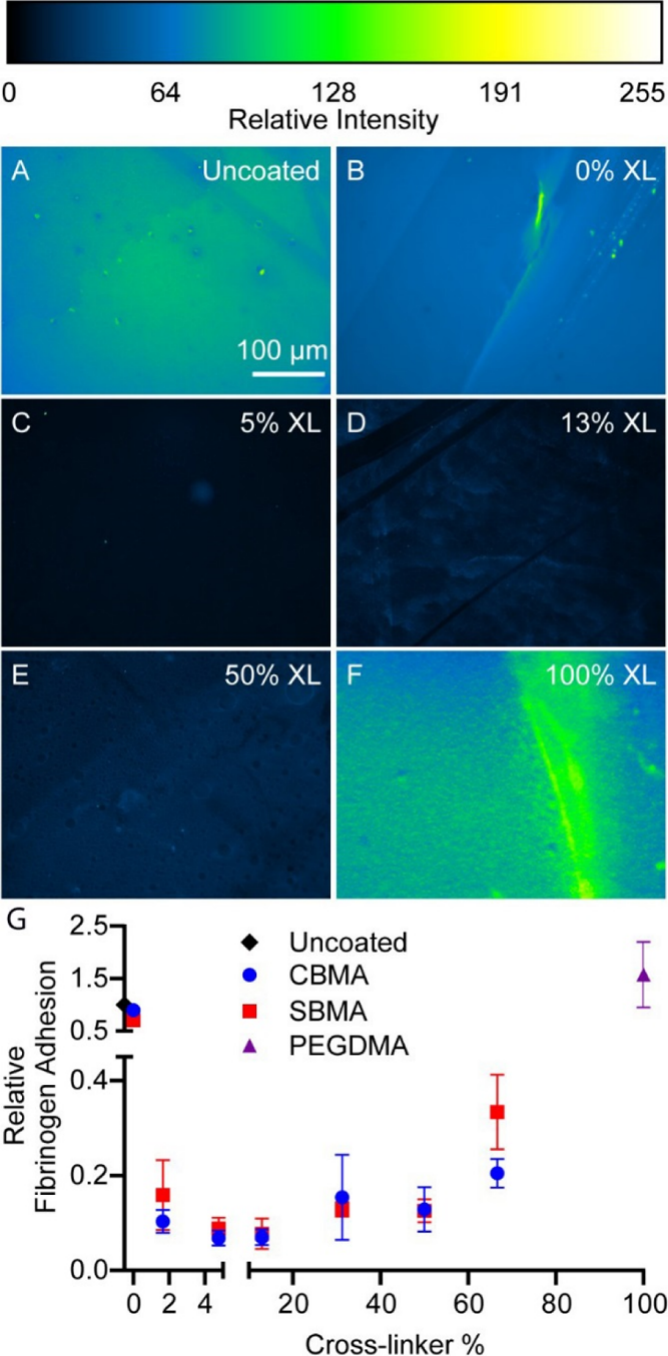
Representative images and relative amount of protein (fibrinogen) adsorption to samples of uncoated and coated PDMS. Images show protein adhesion to (A) an uncoated sample and CBMA films with (B) 0, (C) 5, (D) 13, (E) 50, and (F) 100% cross-linker. (G) Fibrinogen adsorption relative to uncoated PDMS as determined by fluorescence for samples of PDMS coated with CBMA (blue) and SBMA (red) as a function of the cross-linker (PEGDMA). Three samples were analyzed at each cross-link density. The error bars represent the standard error of the mean.

Fibrinogen adsorption was measured and normalized to that of uncoated PDMS, as shown in Figure 1, as a function of cross-linker percentage. Figure 1A–F shows representative images of CBMA thin films with different cross-linker percentages using a heat map to convey the relative fluorescence intensity from adsorbed fibrinogen. These images highlight the significant reduction of fibrinogen in the 5% cross-linker sample (Figure 1C), particularly when compared to uncoated PDMS (Figure 1A). The images also demonstrate that incremental increases in the cross-linker over the 5–50% range led to subtle increases in fibrinogen adhesion (Figure 1D,E) while still maintaining resistance to biofouling relative to uncoated films. Interestingly, the zwitterionic thin films grafted without any cross-linking allowed significantly more fibrinogen adsorption than the cross-linked samples. As indicated in Figure 1G, for both CBMA and SBMA films, at relatively small amounts of cross-linker, the fluorescence decreased substantially when compared to both uncoated PDMS and films without cross-linking. A 20-fold reduction in protein adsorption was observed at approximately 5 and 13 wt % cross-linker. As the cross-link density was increased, small increases in fluorescence occurred with compositions of up to 50% cross-linker still resulting in an order of magnitude decrease in fibrinogen adhesion compared to uncoated PDMS. The zwitterions appeared to contribute strong antifouling character to the thin film, even with significant amounts of cross-linker. With 67% cross-linker, SBMA and CBMA behavior diverged to some degree, with the SBMA films showing roughly double the amount of protein adhesion relative to CBMA. This finding may indicate that CBMA polymer chains reduce protein adhesion more effectively at higher cross-link densities, perhaps due to CBMA more effectively directing the surrounding water molecules into stabilized hydrogen-bonding networks.^31^

When no zwitterionic monomers were included within a neat PEGDMA film, fibrinogen adhesion increased significantly to a greater degree than that seen on uncoated PDMS, as demonstrated in Figure 1F. Interestingly, 0% cross-linker (Figure 1B) resulted in fibrinogen adhesion roughly equal to that seen on an uncoated surface. Additionally, the 1% cross-linker system demonstrated fibrinogen adhesion at twice the levels of 5% cross-linker. One possible explanation for this behavior is the active surface rearrangement of PDMS.^32,33^ If grafted polymers are linear and not connected to a network independent of the PDMS surface, these rearrangements may result in some, if not all, of the zwitterionic character disappearing from the surface as the grafted polymer is incorporated in the bulk PDMS.^34^ However, when sufficiently cross-linked and grafted to the PDMS surface, any rearrangements would have minimal impact, with an intact zwitterionic thin film that can more effectively prevent protein adsorption.

### Cell Adhesion

Macrophages and fibroblasts are two key inflammatory and synthetic cells in the FBR. Macrophages represent the dominant infiltrating cells in the intermediate events of the FBR and aid in cell signaling/recruitment, removal of foreign debris, and wound healing.^4,36^ Fibroblasts produce extracellular matrix proteins in response to profibrotic signals and contribute to the formation of a dense fibrotic capsule.^35^ Cell adhesion is a critical measure of the ability of a biomaterial to resist biofouling. Thus, macrophages and fibroblasts were selected to determine how cell adhesion is altered by changes in thin-film cross-link density. Identifying a range of cross-link densities that results in lower cell adhesion has the potential to reduce the FBR and its sequelae, including fibrosis at the biomaterial–tissue interface.

**Figure 2:**
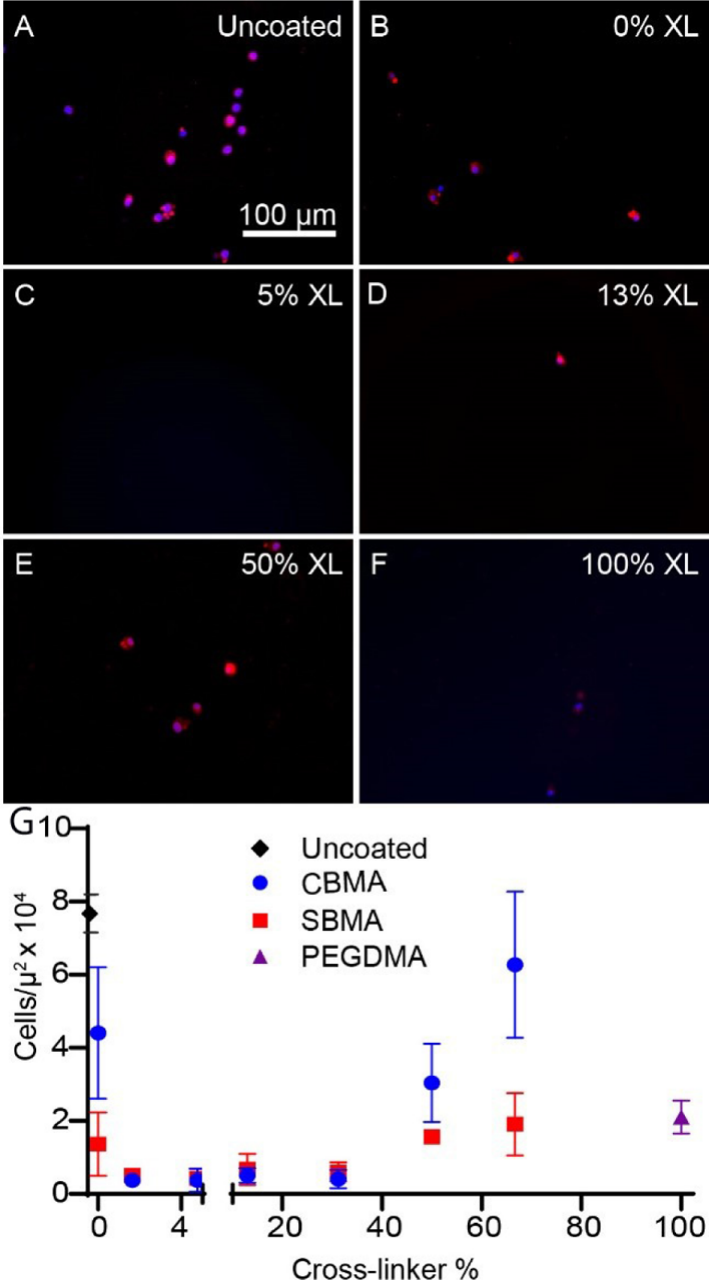
Effect of thin-film cross-link density on macrophage adhesion. Images represent macrophages labeled with anti-F4/8 antibody (red) and nuclei labeled with DAPI (blue) on (A) uncoated PDMS and PDMS coated with CBMA with (B) 0, (C) 5, (D) 13, (E) 50, and (F) 100% PEGDMA cross-linker. (G) Three samples were analyzed at each cross-link density. The error bars represent standard error of the mean. Both CBMA (blue) and SBMA (red) thin films dramatically reduced the fibroblast adhesion compared to uncoated PDMS. Macrophage density was reduced across a broad range of cross-link densities and was lowest in the range of 1.6–31% cross-linker. Cell density increased at lower (0%) and higher (>50%) cross-link densities.

Zwitterionic thin-film coatings on PDMS were created as described above with varied concentrations of the cross-linker. The macrophage density on SBMA and CBMA thin films was measured and compared to that of uncoated PDMS with results plotted in Figure 2 as a function of the cross-linker percentage. Macrophage cell density was reduced as much as 30 times on SBMA and CBMA thin films relative to uncoated PDMS. Significant reductions in cell adhesion were identified on CMBA films within the ranges of 1–50% cross-linker, as shown in Figure 2C–E, especially when compared to uncoated PDMS (Figure 2A). For both SBMA and CBMA systems, the most significant reduction in the macrophage density occurred when using an intermediate range of cross-linker (1–31 wt % cross-linker). Figure 2C,D shows markedly reduced macrophage adhesion on CBMA films with 5 and 13% cross-linker relative to cross-link densities outside of this range. The macrophage density was also reduced on films with no cross-linker present, as depicted in Figure 2B, but not to the level of the lower cross-link density systems, similar to the results observed for fibrinogen adhesion. For both the SBMA and CBMA films, macrophage density began to increase at higher cross-link densities to levels much more on the order to that of uncoated PDMS. At intermediate cross-link densities, SBMA and CBMA seemed to reduce macrophage density to a similar degree. However, in contrast to results with fibrinogen, SBMA thin films prevented cellular adhesion more effectively than the CBMA systems as the cross-link density increased.

**Figure 3:**
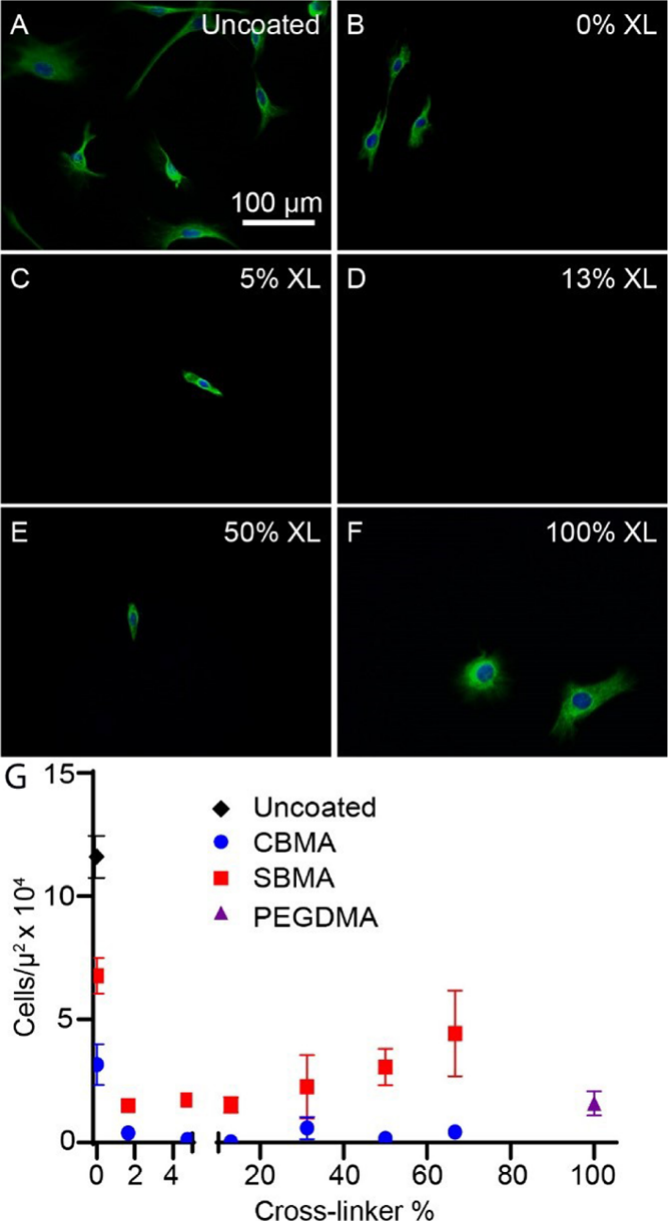
Effect of thin-film cross-link density on fibroblast adhesion. Images represent fibroblasts labeled with antivimentin antibody (green) and nuclei labeled with DAPI (blue) on (A) uncoated PDMS and PDMS coated with a 35% wt monomer solution of CBMA with (B) 0, (C) 5, (D) 13, (E) 50, and (F) 100% PEGDMA cross-linker. (G) Three samples were analyzed for each cross-link density. The error bars represent the standard error of the mean. The lowest cell density occurred in the range of 1.6–31.3% PEGDMA cross-linker. Both CBMA (blue) and SBMA (red) thin films dramatically reduced the fibroblast adhesion compared to uncoated PDMS. A wide range of cross-link densities effectively reduced the fibroblast adhesion. A range of cross-link densities from 1.6 to 13% seemed to provide the strongest antifouling properties. Reduced antifouling effects were seen outside of this range with lower (0% cross-linker) and higher (31.3–66.7%) cross-link densities.

To determine if the low levels of macrophage adhesion were also observed for other important cells in the FBR, cochlear fibrocytes were cultured on thin films. Representative images of the fibrocytes on CBMA thin films with variable cross-link densities are presented in Figure 3. A significant reduction in fibrocyte adhesion was noted on the thin films with intermediate cross-link densities, relative to uncoated PDMS (Figure 3A). For example, Figure 3C–E shows a dramatic reduction in fibrocyte adhesion on CBMA thin films with cross-link densities ranging from 5 to 50%. The fibrocyte density was reduced over 10-fold on the SBMA and CBMA thin films relative to uncoated PDMS. In the SBMA systems, cell density was greater at 0 wt % cross-linker relative to thin films in the range of 1–50% cross-linker. CBMA showed consistent reduction in cellular adhesion across cross-link densities. This reduction was over 10-fold for all cross-linked CBMA films. When comparing SBMA and CBMA systems, SBMA thin films had significantly higher cell densities relative to CBMA at all cross-link densities with greater divergence particularly at the lower and higher cross-link densities. This suggests that CBMA is much more effective for reducing fibroblast adhesion.

The increase in cell density with increasing cross-linker concentrations suggests that increasing cross-linker percentage reduces the antifouling properties of the thin films to some degree. This finding is reasonable, as increasing cross-link density increases the concentration of nonzwitterionic moieties relative to zwitterionic moieties within the polymer network. With a higher density of cross-linking molecules and fewer zwitterionic molecules in the polymer network, a reduced degree of hydration is likely, as well as a reduction in the organization of water molecules in the hydrogel system. This scenario could lead to a biomaterial surface on which the associated water molecules do not cover the surface as uniformly or with as much structure. Thus, subsequent protein adsorption and cell adhesion may occur more frequently. This trend is supported by work elsewhere, in which the grafting density of zwitterionic brushes was varied. Intermediate and higher grafting densities resulted in a more uniform, well-defined zwitterionic layer.^36^ In our study, we found that cross-link densities in the range of 1–50 wt % provided a more consistent hydrogel layer for improved resistance to biofouling. An increase in cell density with no cross-linker was also observed, which could be due to inadequate structural/integral support provided by the cross-linker, resulting in potential surface rearrangements and a less-defined thin-film layer. To verify that these effects could not be further altered with varying grafted hydrogel thickness, fibroblast adhesion was examined at three different film thicknesses, ranging from approximately 20 to 80 μm. As shown in Supporting Information, Figure S3-Sn, the thickness does not change the final cell adhesion.

Together, these results suggest that antifouling properties appear reasonably conserved across a wide range of cross-link densities and may be best achieved using a low-to-intermediate range of cross-link density. The effectiveness of antifouling for protein, fibroblasts, and macrophages is lowest in the approximately 1–50% cross-linker range. Interestingly, while both SBMA and CBMA prevent adhesion of both protein and cells, the material that results in the greatest reduction varies with both protein and fibroblasts adhering less with CBMA and macrophages showing reduced adhesion with SBMA.

### Material Properties

In combination with the antifouling properties of zwitterionic films, an understanding of cross-link density effects on material properties is critical for the ultimate application and durability of the grafted thin films. The cross-link density in a hydrogel system exhibits a marked effect on both the equilibrium swelling and compressive modulus with increased cross-link density leading to a decrease in swelling^37−40^ and an increase in modulus.^41,42^ In order to develop a functional zwitterionic coating that resists biofouling, the hydrogel must be sufficiently durable to withstand the implantation, during which the film may be inserted through both hard and soft tissue while potentially losing water. Additionally, the system must withstand long-term exposure to biological fluids. Typically, durability is increased at a higher modulus, which is achieved with greater cross-linking. On the other hand, as shown above in Figures 1, 2, 3, the greatest reductions in adhesion were shown between 1 and 50 wt % cross-linker, suggesting that greater amounts of cross-linker may compromise antifouling even with potentially greater material stability. To determine the relationship between cross-link density and material properties, both equilibrium swelling and compressive modulus were examined to further identify cross-link densities with an appropriate balance between antifouling and material properties.

**Figure 4:**
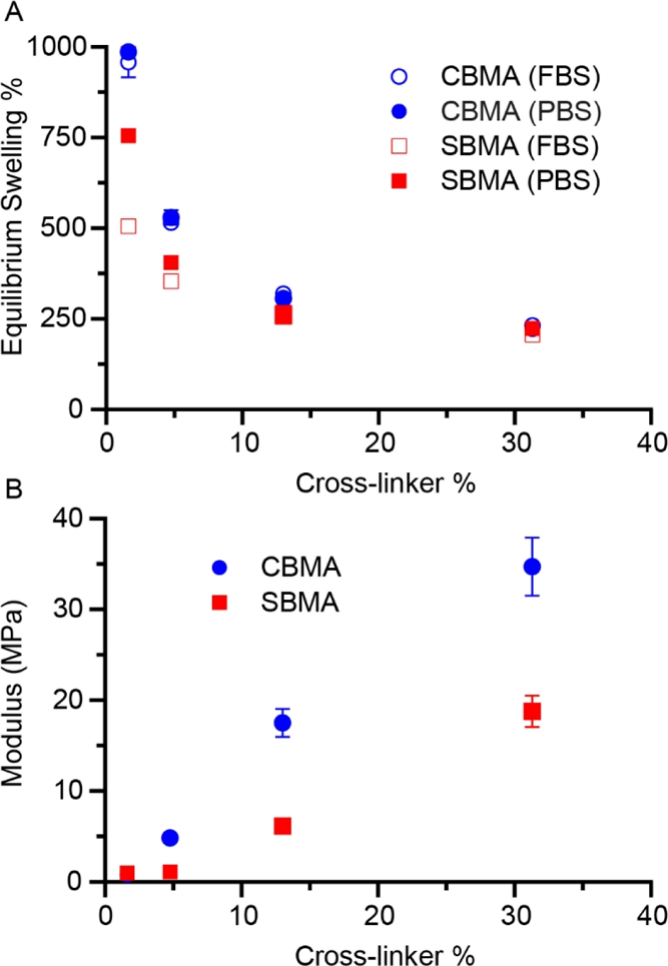
(A) Equilibrium swelling percentage and (B) compressive modulus for CBMA and SBMA hydrogels as a function of increasing cross-linker percentage. (A) For equilibrium swelling, samples were soaked in either bovine serum (FBS, open circles) or PBS solution, and compressive modulus was measured for hydrogels swollen to equilibrium in PBS. Each point represents *n* = 3 (A) or *n* = 4 (B) with error bars representing one standard deviation.

Several material characteristics are critical for appropriate zwitterionic hydrogel coating. First, the degree of equilibrium swelling must be great enough to allow sufficient water to enable the inherent zwitterion/water interactions that provide antifouling capacity. At the same time, excessive swelling could significantly increase the overall size of the implant and cause tissue damage while decreasing function. For example, a CI must fit within the scala tympani while minimizing intracochlear trauma. Additionally, large degrees of swelling may decrease the mechanical stability^43^ and adhesion to the PDMS substrate. In order to understand the impact of cross-link density of zwitterionic thin films on swelling, the equilibrium water uptake of zwitterionic hydrogels as a function of cross-link percent was examined in PBS and FBS (Figure 4A). As expected for hydrogel systems, the equilibrium swelling decreased with the increasing amount of cross-linker in the zwitterionic hydrogels. At the lowest cross-link density in PBS, CBMA hydrogels showed a 30% greater fluid uptake than SBMA hydrogels. CBMA hydrogels consistently swelled to a greater degree. At higher concentrations of cross-linker, the difference became statistically insignificant. CBMA hydrogels in PBS swelled roughly 4 times the amount between the lowest and the highest amount of cross-link density. In comparison, SBMA swelling was approximately 3 times greater at the lowest cross-link density when compared with the highest. As the amount of cross-linker in a system increases, the network formed becomes denser with greater numbers of connecting bonds. The resulting polymer network thus has decreased free volume, enabling water uptake.^44^ Equilibrium swelling approached an asymptote around a cross-linker concentration of 10%, approaching the equilibrium swelling of neat PEGDMA. While the hydrogels do swell significantly, as shown in Figure 4, no delamination was observed for the photoinitiation conditions as reported, indicating adequate covalent grafting between the PDMS and the zwitterionic hydrogel.^21^

To mimic biological conditions where implant surfaces are exposed to serum fluids and proteins, hydrogel equilibrium swelling was also assessed in FBS. When the hydrogels were soaked in serum, the general trends remained the same. The greatest swelling occurred at low cross-link densities, and values at the highest cross-link density were not significantly different from samples swollen in PBS. The samples soaked in serum reached a lower equilibrium swelling percent than samples in PBS, especially for the two lowest cross-link densities of SBMA. Jiang and colleagues noted that although SBMA attracts more overall water molecules, CBMA attracts the water molecules more strongly with greater organization. The most significant differences in water binding were attributed to the anionic moieties, whereas the cationic moieties showed similar effects.^17^ When proteins are introduced to the system from FBS, the negative charges of the proteins may interact with the cationic ions of the zwitterions and lead to charge shielding. Thus, stronger interactions between water and CBMA may lead to less change in swelling in FBS. Conversely, proteins may be able to interact to a greater degree with SBMA interfering with greater water association. Additionally, nonspecific protein adsorption to CBMA and SBMA has been examined in varying concentrations of serum. While relatively low adsorption (even at high protein levels) was observed on CBMA polymers, large increases in adsorption on SBMA polymers were observed with greater protein concentration. Since the distance between ions is greater in SBMA, the hydration layer is less uniform and not very strong.^45^ The greater swelling difference between SBMA in FBS and PBS is likely due to these intermolecular interaction differences. CBMA interacts with water to a greater extent, which is demonstrated by the greater amount of swelling at low cross-link densities and likely contributes to the greater antifouling effect for most systems in comparison to SBMA.

With greater amounts of cross-linker and higher cross-link density, mechanical stability, including the compressive modulus of hydrogels, should also increase. However, if too much cross-linking occurs, the hydrogel will become brittle and more easily damaged. The effect of cross-linker percentage on compressive modulus in the zwitterionic hydrogel systems is shown in Figure 4B. As expected, the compressive modulus increased for swollen stand-alone CBMA and SBMA hydrogels as the percentage of cross-linker increased. Interestingly, a 20-fold increase in compressive modulus was observed from the lowest amount of cross-linker to the highest for SBMA, with a 50-fold increase for CBMA. Both CBMA and SBMA hydrogels experienced monotonic increases over the examined range of the cross-linker. CBMA exhibited a higher modulus than SBMA with the most cross-linked CBMA hydrogel showing a modulus nearly double that of the SBMA hydrogel perhaps due to the stronger ionic interactions between CBMA polymers.^46^

Swelling and compressive modulus were tested for monomer solutions with different water levels.^21^ To allow for the exploration of a greater range of cross-link densities, differences in the water content were necessary to obtain monomer solutions which would not phase-separate upon polymerization. Swelling behavior between the lower (35%) and higher (52.5%) water content formulations was quite different. When formed with less water, hydrogels swelled to a much lower extent (Figure S1-Sn(A)). Swelling of CBMA hydrogels plateaued at about the same cross-linker percent as with systems polymerized with the higher amount of water, while SBMA reached a lower amount of overall swelling. This behavior is most likely due to the smaller and decreased number of pores formed in hydrogels with less initial water. No statistically significant difference was noted in the compressive modulus between the SBMA hydrogels formed with varying amounts of water (Figures 4B and S1-Sn(B)).

These results suggest that CBMA hydrogels should exhibit greater durability. Moreover, CBMA usually demonstrates greater antifouling capabilities, as shown in previous sections and other studies.^21,47^ The antifouling and durability advantages of CBMA can likely be attributed to its enhanced intermolecular interactions.^17^ Due to the stronger interactions between CBMA molecules, a higher compressive modulus is obtained even with greater swelling. CBMA also shows greater affinity for the organization of the water molecules, leading to a greater swelling and a stronger hydration layer at the surface,^45^ which likely decreases biofouling on CBMA hydrogels compared to SBMA hydrogels.

**Figure 5:**
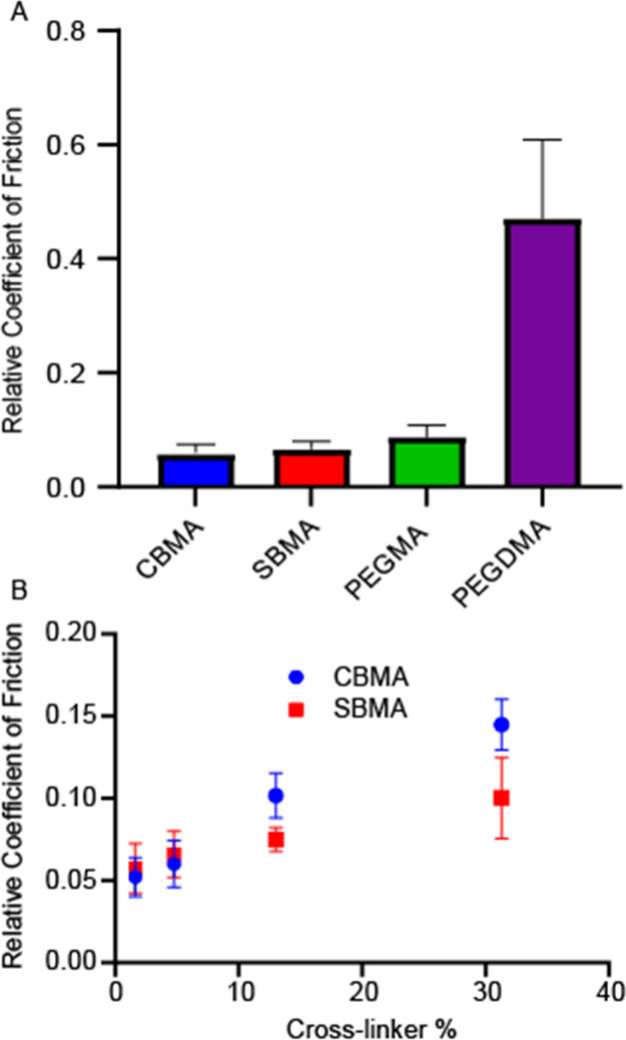
Coefficient of friction for hydrogels normalized to uncoated PDMS (standard deviation = 0.0430) including (A) different zwitterionic and PEG hydrogels with 4% cross-linker and (B) for zwitterionic hydrogels, CBMA and SBMA, as a function of cross-linker percent. For each bar or point, *n* = 4 with the standard error of mean reported for each set as normalized to PDMS.

The absorption of water by hydrogels not only affects bulk material characteristics but also dramatically alters the surface properties.^48^ One surface property that plays a significant role in the implantation of many biomaterials is the coefficient of friction. This measure of lubricity is a dimensionless number that scales the force between two objects (friction) and the force holding them together.^49,50^ Decreasing the coefficient of friction for the surface of an implant will decrease the insertion resistance, leading to decreased trauma to the insertion site.^51^ To compare the effect of zwitterionic monomers in hydrogels on the coefficient of friction, tribometry was used to measure the relative coefficient of friction for uncoated PDMS and PDMS photografted with CBMA, SBMA, and PEGMA hydrogels with 4% PEGDMA cross-linker in comparison to photografted PEGDMA hydrogels (Figure 5). The coefficient of friction for each of the coatings was normalized to that observed on uncoated PDMS. CBMA-, SBMA-, and PEGMA-grafted thin-film hydrogels all exhibited similar relative coefficient of friction values, showing an overall 10-fold decrease from that of PDMS. The coefficient for CBMA films was slightly lower than that of SBMA, while PEGMA films showed the highest coefficient among the three, approximately double that of CBMA films. On the other hand, the coefficient of friction for a cross-linked PEGDMA network without any monomethacrylate monomers was about half that of uncoated PDMS (Figure 5A). These results show clearly that the inherent coefficient of friction for the neat cross-linked PEGDMA is much greater than that of the zwitterionic materials. It is reasonable to believe that the addition of this cross-linker in zwitterionic hydrogel systems induces increases in the coefficient of friction, significantly decreasing the surface lubricity.

To further elucidate the effect of cross-linking on the lubricity of zwitterionic hydrogels, relative coefficient of friction was determined for CBMA and SBMA films with different cross-link densities. The relative coefficient of friction for both CBMA- and SBMA-grafted PDMS decreased up to 20 times relative to uncoated PDMS, as shown in Figure 5B. An upward trend in the coefficient of friction was observed with increasing cross-link density for both CBMA and SBMA films. At a lower cross-link density, CBMA hydrogels appeared to have a slightly lower coefficient of friction, with lower coefficients of friction observed for SBMA at higher cross-link densities. CBMA hydrogels showed a greater overall change in the coefficient of friction and thus a greater dependence on the amount of cross-linker. This behavior is likely also due to the greater interactions within the CBMA hydrogel and with water, which become less prominent as the amount of PEGDMA and cross-link density increases.

Both photografted CBMA and SBMA hydrogels showed significantly decreased coefficients of friction relative to PDMS and PEGDMA, with the greatest reduction noted at lower cross-link densities. While antifouling is also greatest with a lower amount of cross-linker, other mechanical properties (swelling and compressive modulus) improve as the cross-link density increases. Desirable antifouling properties and low coefficient of friction properties at low cross-link densities must be balanced with the greater durability and compressive modulus observed at higher cross-link densities. This work shows that this balance can be achieved at intermediate cross-linker concentrations ranging from 1 to 50%, with the best antifouling usually in the range of 5–15%. Compressive modulus and swelling properties both become more amenable for implant coatings with more cross-linkers, suggesting that the cross-linker should be kept as high as possible without compromising antifouling. For example, compressive modulus shows significant increases at 12% cross-linker, the concentration at which swelling begins to plateau. Adhesion studies of protein and cells demonstrated low adhesion with cross-linked films up to approximately 12% cross-linker. Thus, at this cross-link density, a reasonable combination of mechanical durability and antifouling could be achieved. By understanding the contrasting impacts of cross-link density on both mechanical properties and antifouling characteristics, grafted thin-film hydrogels have been created with appropriate material properties and significant resistance to protein and cellular adhesion.

## Conclusions

Zwitterions show promise in reducing the FBR when grafted to the surface of implanted biomaterials by altering surface chemistry and dramatically reducing protein and cellular adhesion. The mechanical and antifouling properties of photografted zwitterionic thin films change with thin-film cross-link density. However, the antifouling properties appear conserved across a wide range of cross-link densities, with significant reductions in protein and cell adhesion of fibroblasts and macrophages observed. This wide range of cross-link densities allows opportunities to tune the mechanical properties (i.e., modulus, coefficient of friction, and swelling) to generate robust films that are mechanically stable, yet also able to resist biofouling. Photografted thin films with adequate antifouling properties and sufficient mechanical properties have the potential to reduce the FBR in a variety of biomaterial applications.

## Supporting Information Available

SEM images of uncoated PDMS and PDMS coated with CBMA and SBMA; confocal microscopy images depicting thicknesses of thin films as a function of cross-linker percentage; and fibroblast cell density on SBMA films with a variable thin-film thickness (PDF)
