# Supplementary material for: Antifouling and Mechanical Properties of Photografted Zwitterionic Hydrogel Thin-Film Coatings Depend on the Cross-Link Density
Source: ACS Biomater Sci Eng. 2021 Aug 4;7(9):4494–502. doi: 10.1021/acsbiomaterials.1c00852 (PMC8441969; doi:10.1021/acsbiomaterials.1c00852)
Supplement: Supplementary file 1 — ab1c00852_si_001.pdf [file ab1c00852_si_001.pdf]

TITLE. Antifouling and mechanical properties of photografted zwitterionic hydrogel thin film coatings depend on cross-link density

AUTHOR NAMES. Megan J. Jensen[1], Adreann Peel[2], Ryan Horne[1,2], Jamison Chamberlain[2], Linjing Xu[1], Marlan R. Hansen[1,3,4], C. Allan Guymon[2*]

Number of pages: 4

Number of figures: 3

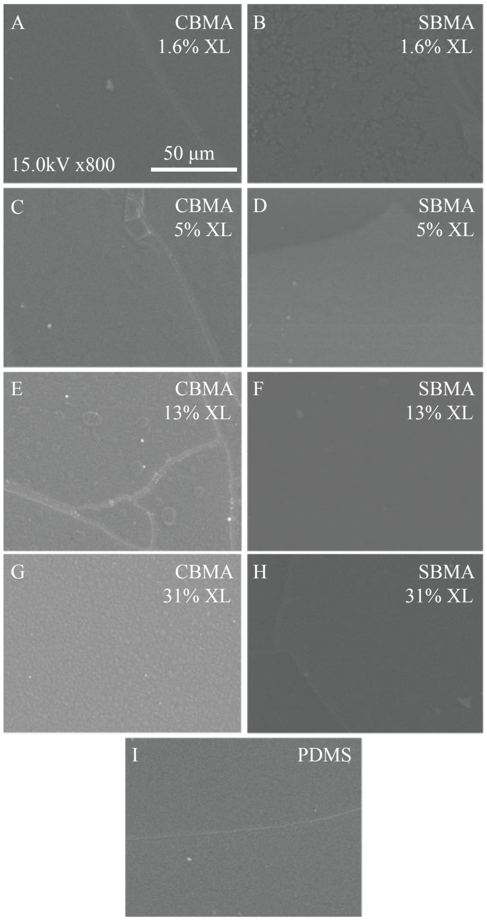

Figure S1-Sn. Surface scanning electron microscopy images at 800x magnification produced with an excitation beam accelerated at 15.0 kV. CBMA thin films on PDMS are pictured in the left column (A, C, E, and G) and SBMA thin films on PDMS in the right column (B, D, F, and H) along with an uncoated PDMS (I) control. The surface of the films is generally observed to be regular, smooth, and featureless. Minor salt deposits from the storage in phosphate buffered saline are visible in some micrographs (e.g. E and I).

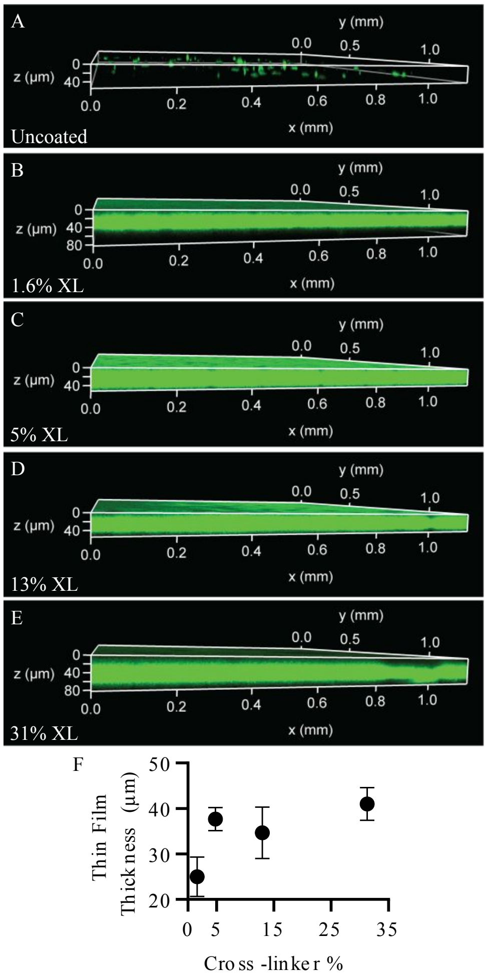

Figure S2-Sn. Representative confocal microscope images of uncoated medical-grade PDMS (A), and thin films of SBMA (B-E) with variable cross-linker percentages (% XL). 10 mg/mL sodium fluorescein solution in phosphate buffered saline was used as a water tracer to illuminate the geometry of the hydrogel. The thicknesses of thin films (n=3) as a function of cross-linker % are plotted (F). Above a threshold of ~2-3% cross-linker, thin film thickness does not vary significantly (~38 µm). Lower cross-linker percentages yield thinner films (~25 µm), likely as these films are more compressible in the confocal microscope environment. However, these variations are relatively minor and are unlikely to be driving differences in anti-fouling behavior.

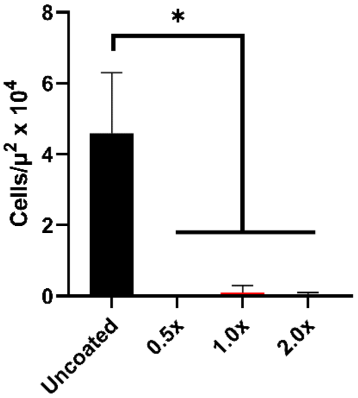

Figure S3-Sn. Fibroblasts (3T3 cells) were cultured on uncoated medical-grade PDMS and compared to SBMA thin films of 0.5x (~19 μm), 1.0x thickness (~38 μm, also the thickness used throughout this work), and 2.0x (~76 μm). The thickness of these coatings were manipulated by adjusting the amount of prepolymer solution placed on the surface. While all thin films demonstrated significant reductions in fibroblasts (*$p<0.05$), no significant difference in cell adhesion was noted between coating groups ($p>0.05$). These results give evidence that film thickness does not modulate the anti-fouling ability of the thin films over a wide range of thicknesses.
